# Atypical Presentation of Thrombotic Thrombocytopenic Purpura without Hematological Features

**DOI:** 10.18502/ijhoscr.v14i3.3724

**Published:** 2020-07-01

**Authors:** Balraj Singh, Kok Hoe Chan, Parminder Kaur, Varun Modi, Michael Maroules

**Affiliations:** 1 Department of Hematology/Oncology, Saint Joseph University Medical Center, Paterson, New Jersey, United States; 2 Department of Medical Education, Saint Michael's Medical Centre, New York Medical College, New Jersey, United States; 3 Orange Regional Medical Centre, New Jersey, USA

**Keywords:** Thrombotic thrombocytopenic purpura (TTP), ADAMTS 13, Microangiopathic hemolytic anemia, Thrombocytopenia

## Abstract

Thrombotic thrombocytopenic purpura (TTP) is a life-threatening disease, usually diagnosed with high index of suspicion. The pathophysiology of TTP is due to severe deficiency of von Willebrand factor cleaving protease, known as ADAMTS 13. Early diagnosis is crucial as without treatment TTP is associated with high mortality rate. Plasma exchange is currently the mainstay of treatment. Nonetheless, the classical pentad of microangiopathic hemolytic anemia (MAHA), thrombocytopenia, neurological dysfunction, kidney dysfunction and fever are seen only in 40 percent of the patients. MAHA and thrombocytopenia are the common presenting features. Presentation with thrombotic complication without hematological features (MAHA and thrombocytopenia) is rare and makes the diagnosis difficult. Herein, we report an unusual presentation of a 53-year-old male, who was initially presented in 2014 with classical features of TTP, however had an atypical presentation of TTP in 2016 with only neurological features without hematological features.

## Introduction

 Thrombotic thrombocytopenic purpura (TTP) is classically characterized by the pentad of MAHA, thrombocytopenia, neurological dysfunction, kidney dysfunction and fever. However, only 40 percent of patients have all the five manifestations^1^. A high index of suspicion is the key to the early diagnosis of TTP. TTP is caused by decreased activity of the plasma metalloproteinase ADAMTS 13 (A Disintegrin And Metalloproteinase with a Thrombospondin type 1 motif, member 13), the key enzyme involved in the cleavage of ultra-large (von Willebrand Factor) VWF multimers into smaller less procoagulant multimers^2^^,^^3^. Micro-thrombosis is composed of platelet and ultra large von Willebrand factor multimers in the small blood vessels, which leads to organ dysfunction^4^^,^^5^. Shearing of red blood cells by the micro-thrombosis will lead to schistocytes and MAHA. 

Thrombocytopenia observed in TTP is secondary to consumption in the micro-thrombosis. Patients commonly presented with clinical features related to the thrombosis of the involved vessels and usually MAHA and thrombocytopenia are present. Thrombosis may involve brain, which presents as stroke, seizures, altered mental status or kidney ischemia or acute coronary syndrome or pulmonary embolism or unusual visual presentation like scotomas, blurred or double vision^6^^-^^8^. Presentation with thrombotic complication without hematological features (MAHA and thrombocytopenia) is rare and makes the diagnosis difficult^9^. We herein report a case of 53-year-old male who initially presented in 2014 with classical features of TTP (MAHA, thrombocytopenia, neurological manifestations), however, had an atypical presentation two years later with only neurological features without hematological features.

## Case presentation

A 53-year-old male with past medical history of TTP (diagnosed in 2014), gout, hypertension, diabetes mellitus, and stage 3 chronic kidney disease presented to the emergency department with complaints of left sided facial weakness and left-hand paresthesia. Symptoms resolved in less than 5 minutes, but it recur again with slurring of speech later. Otherwise, patient denied headache, blurry vision, and loss of consciousness or seizures. Cardiovascular and respiratory exam was normal. Neurological exam was positive for facial asymmetry with angle of the mouth deviated towards the right side and decreased tactile sensation on the left side of face. Otherwise patient was awake, alert, oriented to person, place, and time, followed commands, strength was 5/5 in all 4 extremities without drift. Reflexes were 2+ and symmetric throughout. No dysmetria on finger-to-nose. CT scan was done which showed low-density area in the right anterior corona radiate suspicious for acute infarct. Due to late presentation, low NIHSS (National Institutes of Health Stroke Scale) score and history of TTP, patient was not given thrombolytic therapy. MRI was also done which showed acute infarct in the right parietal lobe with subacute infarcts in the posterior right frontal and left parietal lobes.

Initial blood work on presentation showed a hemoglobin of 11.5 g/dL (normal range: 13.5-17.5), platelets 129 k/mm3 (normal range: 140-440), reticulocyte count 1.8 % (normal range: 0.5- 2), lactate dehydrogenase 213 U/L (normal range: 140-271), and haptoglobin 252 mg/dL (normal range: 34-200). Patient’s baseline creatinine was 1.4 mg/dL (normal range: 0.6-1.3), had an acute kidney injury with creatinine of 1.79 mg/dL, which went back to baseline the following day. Peripheral blood film was negative for schistocytes but given the previous history of TTP and multiple strokes on MRI, plasmapheresis was carried out. ADAMTS 13 activity measured prior of plasmapheresis was less than 10 percent. Patient was improving after plasmapheresis and was discharged with no neurological deficits and to follow up in hematology-oncology clinic. Patient has the ADAMTS 13 antibody activity checked periodically after discharge and was in remission.

Back in 2014, when patient was first diagnosed with TTP, patient was presented to the emergency department for circumoral paresthesia and abdominal ecchymosis. Blood works during that admission showed normocytic normochromic anemia with hemoglobin of 9 g/dL (normal range: 13.5-17.5), platelets 6 k/mm3 (normal range: 140-440), reticulocyte count of 4.3 % (normal range: 0.5- 2), lactate dehydrogenase of 880 U/L (normal range: 140-271), and haptoglobin of less than 10 mg/dL (normal range: 34-200). Peripheral blood film revealed schistocytes, ADAMTS 13 activity less than 10 percent and ADAMTS 13 antibody of 68 U/mL (normal range: less than 12).

## Discussion

 TTP, a subtype of thrombotic microangiopathy, was first described in 1924 by Moschowitz. It was described with a young girl with weakness, pallor, purpura and hemiparesis, died within days, secondary to heart failure. Autopsy showed hyaline thrombosis in small vessels of most organs^10^. TTP can occur, either congenital or acquired. In congenital TTP, multiple mutations will be identified in the ADAMTS 13 gene and the involvement of both alleles will result in a severe deficiency of ADAMTS 13 activity^11^. Acquired ADAMTS 13 deficiency is due to the formation of autoantibodies against ADAMTS 13^12^. The incidence of acquired TTP is much greater in adults (2.9 cases per 1 million per year) than as compared to children (0.1 cases per 1 million per year)^13^. 

Sarcode et al. in a retrospective study of 70 patients have found out that MAHA and thrombocytopenia were present in 100 % of patients, followed by neurologic involvement in 90 %, renal involvement in 50 %, and fever only in 25 % of cases^14^. Neurologic symptoms like severe headaches, seizures, aphasia, syncope, dysarthria, blurred or double vision, altered mental status, paresis, stroke, semi-coma, and coma has also been reported^15^. A high index of suspicion is the key to the early diagnosis of TTP. An acute attack of TTP presenting in the absence of hematological findings of MAHA and thrombocytopenia is rare^9^. Only few cases have been reported in the literature, to our knowledge. Kalish et al. reported 5 cases of TTP presenting with thrombotic event like pulmonary embolism or cerebral vascular accident with no hematological criteria^9^. In the study, two of the patients was presented with cerebral vascular accident and no hematological features, however had positive ADAMTS 13 inhibitors and less than 5 percent ADAMTS 13 activity^9^.

ADAMTS 13 is a dis-integrin-like metalloproteinase, which regulates a key physiological process of coagulation in the circulation by cleaving VWF multimers into smaller less procoagulant multimers^2^^,^^3^. High circulating levels of unusually large VWF multimers (UL-VWFM) have strong procoagulant activity and facilitate platelet adhesion and aggregation^16^. MAHA and thrombocytopenia are results of mechanical hemolysis and consumption, respectively. However, in our patient, there were neurological features without hematologic manifestations of MAHA and thrombocytopenia. One possible reasons for such atypical presentation was that the micro-thrombosis caused by the platelet rich thrombi in the small blood vessels in the brain was not enough to produce MAHA and thrombocytopenia but enough to cause neurological manifestations^17^. 

There was clinical data supporting that high VWF may lead to thrombosis without hematological manifestations of TTP. This was reported by Sonneveld et al. in their meta-analysis, which showed an association between high VWF levels and arterial thrombosis (ischemic stroke and coronary heart disease)^18^. The meta-analysis consisted of 616 cases, it showed that low ADAMTS 13 levels were associated with an increased risk of ischemic stroke. However, these studies were case-control in design and small in sample size. Therefore, prospective cohort studies are in fact required to confirm the role of ADAMTS 13 in the pathogenesis of ischemic stroke^18^. 

Currently, plasma exchange and immunosuppression are the mainstay of treatment for TTP. In 1991, a randomized controlled trial has showed a survival rate of 78 % with plasma exchange^19^. Daily plasma exchange removes the autoantibodies against ADAMTS 13 whereas immunosuppressive treatment acts on the underlying autoimmune process^20^. Recovering platelet count can be used as a guide for deciding the frequency of daily plasma exchange as platelet counts correlates with the disease activity^21^. 

On the other hand, the role of monitoring ADAMTS 13 activity following recovery is still remain controversial. Nonetheless, Page et al. reported that a subset of patients may have severe ADAMTS 13 deficiency (less than 10 percent) during remission after recovery and completely asymptomatic, and this severe deficiency during remission may predict the risk of future relapse^22^^-^^24^. Numerous reports have also advocated the pre-emptive treatment of patient with severe ADAMTS 13 deficiency with Rituximab or other immunosuppressive therapy^25^^,^^26^. However, those study has small in sample size. Thus, the ultimate role of monitoring ADAMTS 13 activity during remission and the pre-emptive treatment of these group of patients with Rituximab still remain unclear and warrant a larger scale randomized control trial or prospective cohort study.

## CONCLUSION

 TTP is a life-threatening disease with high mortality if treatment is not given in a timely manner. A high index of suspicion is required for making the diagnosis. TTP can be present with a thrombotic complication during the initial or recurrent episodes without the hematological features of MAHA and thrombocytopenia. Thus, low level of ADAMTS 13 plays an important diagnostic role especially in atypical cases of TTP. Physician should be aware of the atypical presentations so that the diagnosis can be made in a timely manner.
